# Supplementary material for: Resistin and IL-15 as Predictors of Invasive Mechanical Ventilation in COVID-19 Pneumonia Irrespective of the Presence of Obesity and Metabolic Syndrome
Source: J Pers Med. 2022 Mar 3;12(3):391. doi: 10.3390/jpm12030391 (PMC8955294; doi:10.3390/jpm12030391)
Supplement: Supplementary file 1 [file jpm-12-00391-s001.zip › Suplemmentary material.pdf]

Table S1. Correlations between the baseline levels of cytokines with clinical characteristics of the COVID-19 patients.

Variables	Patients' characteristics							
	**Clinical characteristics**							
	**Age**	**Gender**	**BMI**	**DLP**	**MS**	**Alcohol**	**Smoking**	**Exercise**
RES	n.s.	n.s.	n.s.	n.s.	n.s.	n.s.	n.s.	n.s.
INF-β	0.416*	n.s.	n.s.	n.s.	n.s.	n.s.	n.s.	n.s.
INF-γ	n.s.	n.s.	n.s.	n.s.	n.s.	n.s.	n.s.	n.s.
IL-1β	n.s.	n.s.	n.s.	n.s.	-0.215*	n.s.	n.s.	n.s.
IL-6	n.s.	n.s.	n.s.	n.s.	n.s.	n.s.	n.s.	n.s.
IL-7	n.s.	n.s.	n.s.	n.s.	n.s.	n.s.	n.s.	n.s.
IL-8	0.169*	n.s.	0.198*	n.s.	n.s.	n.s.	n.s.	n.s.
IL-10	n.s.	n.s.	n.s.	0.290*	n.s.	n.s.	n.s.	n.s.
IL-13	-0.190*	n.s.	n.s.	n.s.	n.s.	n.s.	n.s.	n.s.
IL-15	n.s.	-0.212*	n.s.	0.178*	n.s.	n.s.	n.s.	n.s.
IL-17A	n.s.	n.s.	n.s.	n.s.	n.s.	n.s.	n.s.	n.s.
IL-18	n.s.	-0.178*	n.s.	n.s.	n.s.	0.177*	n.s.	n.s.
MCP-1	n.s.	n.s.	n.s.	n.s.	n.s.	n.s.	-0.191*	n.s.
TNF-α	n.s.	n.s.	n.s.	n.s.	n.s.	n.s.	n.s.	-0.217*

	**Symptoms' characteristics**				
	**Symptoms' evolution**	**ICU admission**	**Radiological characteristics**	**Severity of pneumonia**	**Respiratory failure**
RES	0.453**	0.364**	0.241**	0.455**	0.232**
INF-β	n.s.	n.s.	n.s.	n.s.	n.s.
INF-γ	n.s.	n.s.	n.s.	n.s.	n.s.
IL-1β	n.s.	n.s.	n.s.	n.s.	n.s.
IL-6	0.357**	0.333**	n.s.	0.313**	n.s.
IL-7	n.s.	0.174*	n.s.	n.s.	0.177*
IL-8	0.485**	0.324**	0.347**	0.472**	0.476**
IL-10	n.s.	n.s.	n.s.	0.288*	n.s.
IL-13	n.s.	n.s.	n.s.	n.s.	n.s.
IL-15	0.268**	0.289**	0.249**	0.359**	0.206**
IL-17A	n.s.	n.s.	n.s.	n.s.	n.s.
IL-18	0.223**	0.195*	0.259**	0.239**	0.171*
MCP-1	0.296**	0.342**	0.189*	0.343**	0.304**
TNF-α	0.284**	0.305**	0.207*	0.265**	0.254**

	**Oxygen therapy**			
	**Nasal need or oxygen mask**	**HFNC need**	**MV need**	**MV (+ vasopressors or dialysis) need**
RES	0.232**	0.242**	0.376**	0.355**
INF-β	n.s.	n.s.	n.s.	n.s.
INF-γ	n.s.	n.s.	n.s.	n.s.
IL-1β	n.s.	n.s.	n.s.	n.s.
IL-6	n.s.	n.s.	0.309**	0.298**
IL-7	0.177*	n.s.	n.s.	n.s.
IL-8	0.476**	0.276**	0.381**	0.328**

IL-10	n.s.	n.s.	n.s.	0.288*
IL-13	n.s.	n.s.	n.s.	0.175*
IL-15	0.206*	0.260**	0.363**	0.223**
IL-17A	n.s.	n.s.	n.s.	n.s.
IL-18	0.171*	n.s.	n.s.	0.179*
MCP-1	0.304**	0.215**	0.394**	0.323**
TNF-α	0.254**	0.187*	0.245**	0.259**

DLP, dyslipidaemia; BMI, body mass index; MS, metabolic syndrome; RES, resistin; INF, interferon; IL, interleukin; MCP-1, monocyte chemoattractant protein 1; TNF-α, tumor necrosis factor alpha; ICU, intensive care unit; HFNC, high-flow nasal cannulas; MV, mechanical ventilation; n.s., non-significant. Data are expressed as the correlation coefficient rho of Spearman and p-value ($p < 0.05$ was considered statistically significant; *$p < 0.05$, ** $p < 0.01$).

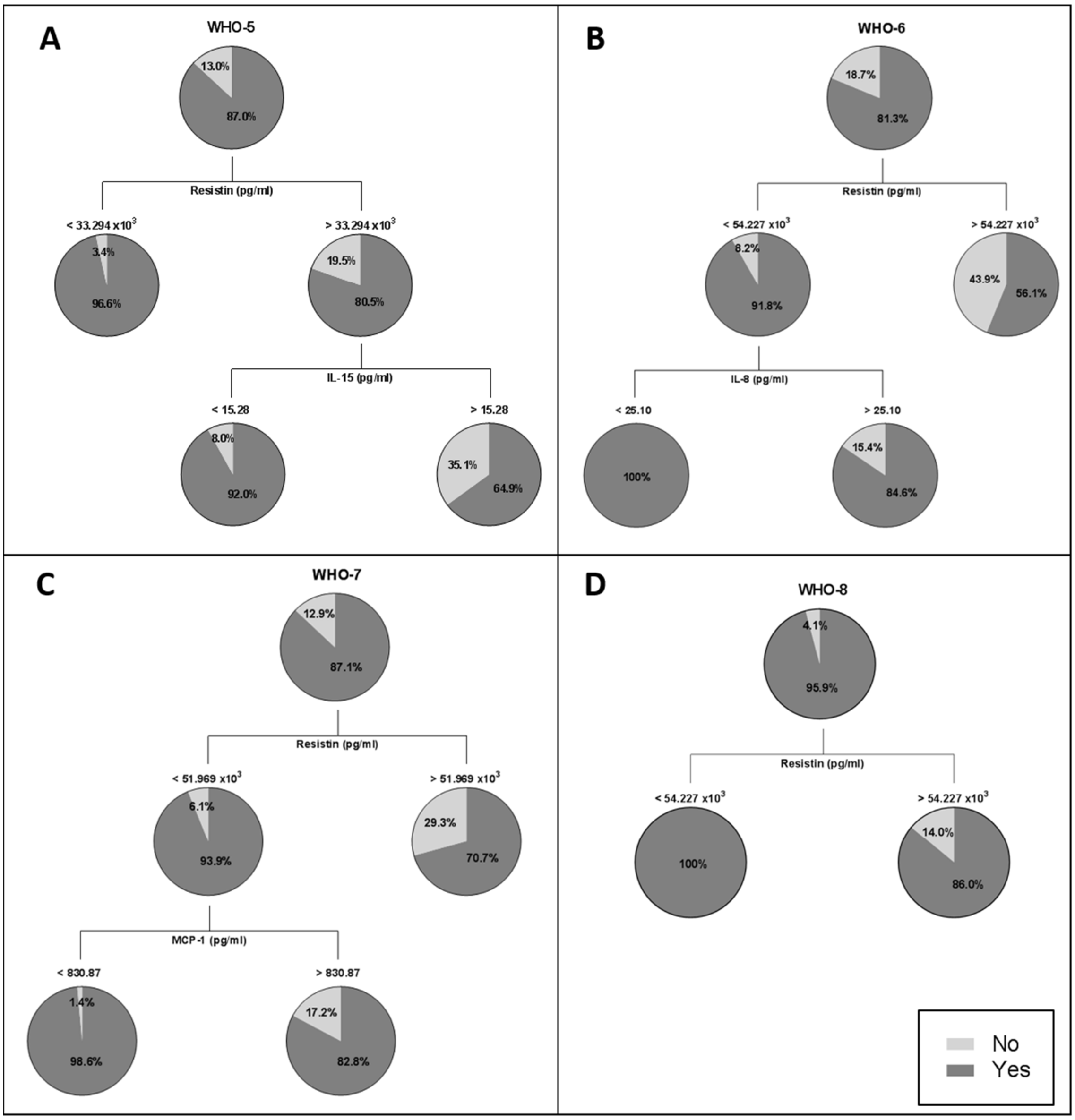

Figure S1. Classification and regression trees of the whole cohort of study were elaborated through CRT method for World Health Organization (WHO) eight-point classification of severe pneumonia by COVID-19 (namely: (5) non-invasive ventilation (NIV) (continuous positive airway pressure (CPAP) or positive bipressure in the airways (BiPAP)) or high-flow oxygen (HFO); (6) intubation with mechanical ventilation (MV) or mask with reservoir; (7) MV or extracorporeal membrane oxygenation (ECMO), support with vasopressors, dialysis/renal replacement therapy; and (8) death. (A) Pie charts represent the proportion of patients who met the WHO 5. (B) Pie charts represent the proportion of patients who met the WHO 6. (C) Pie charts represent the proportion of patients who met the WHO 7. (D) Pie charts represent the proportion of patients who met the WHO 8. IL, interleukin; MCP-1, monocyte chemoattractant protein 1.
